# Prophylactic effect of retromuscular mesh placement during loop ileostomy closure on incisional hernia incidence—a multicentre randomised patient- and observer-blind trial (P.E.L.I.O.N trial)

**DOI:** 10.1186/s13063-023-07089-3

**Published:** 2023-02-01

**Authors:** Sven Müller, Dirk Weyhe, Florian Herrle, Philipp Horvath, Robert Bachmann, Viktor von Ehrlich-Treuenstätt, Patrick Heger, Nadir Nasir, Christina Klose, Alexander Ritz, Anja Sander, Erich Grohmann, Colette Dörr-Harim, André L. Mihaljevic

**Affiliations:** 1 Helios Klinikum Gifhorn, Campus 6, 38518 Gifhorn, Germany; 2 Klinik für Allgemein- und Viszeralchirurgie, Universitätsmedizin Oldenburg, Pius-Hospital Oldenburg, Georgstraße 12, 26121 Oldenburg, Germany; 3 Chirurgische Klinik, Universitätsklinikum Mannheim, Theodor-Kutzer-Ufer 1-3, 68167 Mannheim, Germany; 4 Klinik für Allgemeine, Viszeral- und Transplantationschirurgie, Universitätsklinikum Tübingen, Hoppe-Seyler-Straße 3, 72076 Tübingen, Germany; 5 Klinik für Allgemein-, Viszeral- und Transplantationschirurgie, Ludwig-Maximilians-Universität München, Marchioninistraße 15, 81377 München, Germany; 6 Department of General and Visceral Surgery and Clinical Trial Centre Department of Surgery (ulmCARES), University Hospital Ulm, Albert-Einstein-Allee 23, 89081 Ulm, Germany; 7 Institute of Medical Biometry (IMBI), University of Heidelberg, Im Neuenheimer Feld 130.3, 69120 Heidelberg, Germany; 8 Deutsche ILCO e. V., Nietzschestr. 11, 53177 Bonn, Germany

**Keywords:** Incisional hernia, Surgical mesh, Patient-reported outcome measures, Postoperative complications, Randomised controlled trial

## Abstract

**Background:**

Incisional hernia is a frequent complication following loop ileostomy reversal. Incisional hernias are associated with morbidity, loss of health-related quality of life and costs and warrant the investigation of prophylactic measures. Prophylactic mesh implantation at the time of surgical stoma reversal has shown to be a promising and safe method to prevent incisional hernias in this setting. However, the efficacy of this method has not yet been investigated in a large multicentre randomised-controlled trial (RCT) with adequate external validity. The P.E.L.I.O.N. trial will evaluate the efficacy of prophylactic mesh reinforcement after loop ileostomy closure in decreasing the rate of incisional hernia versus standard closure alone.

**Methods:**

P.E.L.I.O.N. is a multicentre, patient- and observer-blind RCT. Patients undergoing loop ileostomy closure will undergo intraoperative 1:1 randomisation into either abdominal wall closure with a continuous slowly absorbable suture in small-stitch technique without mesh reinforcement (control group) or abdominal wall closure with an additional reinforcement with a retromuscular non-absorbable, macro-pore (pore size ≥ 1000 μm or effective porosity >0%) light-weight monofilament or mixed structure mesh. A total of 304 patients (152 per group) will need to be randomised in the study. Based on inclusion and exclusion criteria, 1,014 patients are expected to be screened for eligibility in order to recruit the necessary number of patients. The primary endpoint will be the frequency of incision hernias within 24 months according to the European Hernia Society definition. Secondary endpoints will be the frequency of surgical site occurrences (including surgical site infections, wound seromas and hematomas, and enterocutaneous fistulas), postoperative pain, the number of revision surgeries and health-related quality of life. Safety will be assessed by measuring postoperative complications ≥ grade 3 according to the Dindo-Clavien classification.

**Discussion:**

Depending on the results of the P.E.L.I.O.N. trial, prophylactic mesh implantation could become the new standard for loop ileostomy reversal.

**Trial registration:**

DRKS00027921, U1111-1273-4657

**Supplementary Information:**

The online version contains supplementary material available at 10.1186/s13063-023-07089-3.

## Administrative information

Note: the numbers in curly brackets in this protocol refer to SPIRIT checklist item numbers. The order of the items has been modified to group similar items (see http://www.equator-network.org/reporting-guidelines/spirit-2013-statement-defining-standard-protocol-items-for-clinical-trials/).

**Table:** 

Title {1}	**P**rophylactic **E**ffect of retromuscular mesh during **L**oop **I**leostomy closure **O**n incisional her**N**ia incidence – a multicentre randomized patient- and observer-blind trial (P.E.L.I.O.N Trial).
Trial registration {2a and 2b}.	DRKS00027921, U1111-1273-4657, date of registration 13th June 2022
Protocol version {3}	1.1; February 12, 2022.
Funding {4}	German Ministry of Research and Education, Bundesministerium für Bildung und Forschung BMBF, Funding number 01KG2023
Author details {5a}	Sven Müller, Helios Klinikum Gifhorn, Campus 6, 38518 Gifhorn, Germany Dirk Weyhe, Klinik für Allgemein- und Viszeralchirurgie, Universitätsmedizin Oldenburg, Pius-Hospital Oldenburg, Georgstraße 12, 26121 Oldenburg, Germany Andre Mihaljevic, Patrick Heger, Nadir Nasir, Colette Doerr-Harim: Department of General and Visceral Surgery and Clinical Trial Centre Department of Surgery (ulmCARES), University Hospital Ulm, Albert-Einstein-Allee 23, 89081 Ulm, Germany Florian Herrle, Chirurgische Klinik, Universitätsklinikum Mannheim, Theodor-Kutzer-Ufer 1-3, 68167 Mannheim, Germany Philipp Horvath, Robert Bachmann: Klinik für Allgemeine, Viszeral- und Transplantationschirurgie, Universitätsklinikum Tübingen, Hoppe-Seyler-Straße 3, 72076 Tübingen, Germany Viktor von Ehrlich-Treuenstätt, Klinik für Allgemein-, Viszeral- und Transplantationschirurgie, Ludwig-Maximilians-Universität München, Marchioninistraße 15, 81377 München, Germany Anja Sander, Alexander Ritz, Christina Klose: Institute of Medical Biometry (IMBI), University of Heidelberg, Im Neuenheimer Feld 130.3, 69120 Heidelberg Erich Grohmann, Deutsche ILCO e. V., Nietzschestr. 11, 53177 Bonn, Germany
Name and contact information for the trial sponsor {5b}	Andre L. Mihaljevic: Department of General and Visceral Surgery, Clinical Trial Centre Department of Surgery (ulmCARES), University Hospital Ulm, Albert-Einstein-Allee 23, 89081 Ulm, Germany Phone: +49 (0) 0731-500-53502 Fax: +49-(0) 731 500-53503 E-mail: andre.mihaljevic@uniklinik-ulm.de
Role of sponsor {5c}	The funding body (German Ministry of Research and Education, Bundesministerium für Bildung und Forschung BMBF, Funding number 01KG2023) has NO role in study design; collection, management, analysis, and interpretation of data; writing of the report; and the decision to submit the report for publication. Furthemore, it has no ultimate authority over any of these activities.

## Introduction

### Background and rationale {6a}

Diverting loop ileostomy is used in colorectal surgery to reduce the consequences of leakage of a bowel anastomosis. According to the Federal Statistical Office of Germany, approximately 15,000 diverting loop ileostomies and 11,500 loop ileostomy closures are performed annually in Germany [1]. The timepoint of ileostomy reversal is debated [2, 3]. Parastomal hernia is frequent after ostomy creation and is estimated to be over 30% by 12 months, 40% by 2 years and 50% or higher at longer duration of follow-up [4, 5]. After primary closure of ileostomy sites, approximately 20% of patients develop an incisional hernia (IH) at the site of stoma reversal within 12 months and up to one-third within 2 years [6] as the incidence of IHs increases over time [7].

Multiple studies have shown that IH causes substantial morbidity and a reduction in the quality of life due to abdominal pain and discomfort, a reduction in the daily activity as well as mortality [8]. Furthermore, IH can cause potentially life-threatening complications with bowel incarceration and strangulation. Furthermore, recurrence of IH following IH repair, which occurs in up to 40% of patients [9], is associated with decreased quality of life [10]. IH causes a substantial burden to healthcare systems worldwide [11, 12]_ and an estimated annual cost of 8.2 million € for the German healthcare system [13].

Because of the important consequences for patients and national healthcare systems, the prevention of incisional hernia has been a subject of intensive investigation over the last decades. For ventral IHs, prophylactic mesh placement at the time of abdominal wall closure is now an accepted technique. Its efficacy has been tested in numerous randomised controlled trials (RCTs) [13–15].

For ostomy reversal, much less evidence is available. Probably because concerns of safety have been voiced regarding an increased risk of surgical site infections (SSI) and mesh infections. Three non-randomised studies have been published on prophylactic mesh placement for ostomy reversal [16–18]. Overall, these three studies showed an overwhelming reduction of IH in the prophylactic mesh group compared to no mesh placement in a systematic review (OR 0.10, 95% CI 0.04–0.27, *p* < 0.001, I2 = 0%, CI 0–91.40%) [19]. However, two of the three studies were retrospective analyses, none of the studies was randomised, follow-up and endpoint assessment were inadequate and studies were at high risk of bias [19]. Furthermore, the technique varied widely between the studies. However, none of the studies showed an increase in the rate of SSI for patients undergoing prophylactic mesh placement at the site of stoma reversal, thus indicating the safety of the technique.

In addition, one recent RCT trial (ROCSS, NCT02238964) compared prophylactic mesh placement in an intraperitoneal onlay position (IPOM) at the time of ostomy reversal to primary closure [20]. In total, 790 patients were 1:1 randomised. The clinically detectable hernia rate, the primary outcome, at 2 years was 12% (39 of 323) in the mesh group and 20% (64 of 327) in the control group (adjusted relative risk [RR] 0.62, 95% CI 0.43–0.90; *p*=0.012). Furthermore, no significant differences were seen in wound infection rate, seroma and serious adverse events, underlining the safety of prophylactic mesh placement. There were, however, considerable differences to the P.E.L.I.O.N trial. First, ROCSS used an industry-sponsored biological absorbable mesh, that is uncommon in hernia surgery and associated with considerable costs. The postulated advantages of a biological mesh over synthetic meshes have so far not been proven in larger controlled trials. On the contrary, a review on mesh selection for ventral hernia repair and incisional hernia prophylaxis draws the conclusion that the use of biological meshes can currently not be recommended [14]. A recent RCT showed that biological meshes are inferior to polypropylene meshes in ventral IH repair [21]. Second, the mesh was placed in an intraperitoneal onlay position (IPOM) rather than in a sublay position as in the P.E.L.I.O.N trial. Third, the primary outcome parameter was IH at 2 years evaluated by clinical exam only. The European Hernia Society (EHS), however, recommends IH evaluation by clinical as well as radiological exams within 24 months. Only a subgroup of patients in the ROCSS study underwent radiological examination and only within a limited follow-up period of 12 months [20]. For this subgroup, results confirmed the findings for the primary endpoint: IH was 20 (9%) of 229 in the prophylactic mesh group vs 47 (21%) of 226 for the control group (adjusted RR 0.42, 95% CI 0.26–0.69; *p*<0.001) [20]. Fourth, ROCSS included all types of stomata (end, loop, colostomy, ileostomy) and not only loop ileostomies as in the P.E.L.I.O.N trial. Finally, the abdominal wall closure in the control group was not standardised nor reported in the trial, raising a relevant concern of a potential bias. Fascial closure technique has an effect on incisional hernia rate, as shown for example by the STITCH trial [22]. In summary, considerable differences exist between ROCSS and the P.E.L.I.O.N trial.

## Objectives {7}

The high rate of IH after loop ileostomy closure with its associated morbidity, loss of health-related quality of life and costs (hernia repair, loss of workforce) warrants the investigation of prophylactic measures. Prophylactic mesh implantation at the time of surgical stoma reversal has shown to be a promising and safe method to prevent IH formation in this setting. However, the efficacy of this method has not yet been investigated in a large multicentre RCT with adequate external validity. P.E.L.I.O.N will fill this evidence gap. The aim of the PELION trial is to evaluate the efficacy of prophylactic mesh reinforcement after loop ileostomy closure in decreasing the rate of IH versus standard closure alone.

## Trial design {8}

P.E.L.I.O.N is a randomised controlled observer- and patient-blinded multicentre surgical superiority trial with two parallel study groups.

## Methods: participants, interventions and outcomes

### Study setting {9}

The study will be carried out in 10 high-volume academic hospitals in Germany. A list of study sites can be found in Supplement 1.

### Eligibility criteria {10}

#### Inclusion criteria for patients

To obtain a homogenous but still representative patient population for analysis and high-external validity, all patients with primary loop ileostomy closure and without further interventions or conditions to the abdominal wall, which might interfere with the primary outcome measure and reduce the reproducibility and interpretability of the data, are eligible for inclusion and will be informed about the P.E.L.I.O.N. trial:

1. Planned elective loop ileostomy closure
2. Adult patients (≥ 18 years of age)
3. Life expectancy > 2 years
4. Written informed consent
5. Ability to understand the character and individual consequences of the clinical trial

#### Exclusion criteria for patients

1. American Society of Anesthesiologist (ASA) physical status class ≥ 4
2. Infected/septic surgical site (risk for surgical site occurrences (SSO) of grade 4 according to Ventral Hernia Working Group (VHWG) classification) (see Supplement 2)
3. Presence of parastomal hernia in loop ileostomy site with fascia defect > 8cm (Table 1)
4. Presence of a concomitant incisional hernia that impedes loop ileostomy reversal or placement of the mesh at the ileostomy site (Table 1)
5. Patients with prior mesh placement on site of ileostomy
6. Chronic renal failure under haemodialysis/peritoneal dialysis
7. Patients under strong immunosuppression or other medications likely to impede wound healing as judged by the operating surgeon. Examples of strong immunosuppression are:
  Steroid therapy ≥ 10 mg/day
  Therapy with antitumor necrosis factor α (anti-TNF-α) within the last 4 weeks
  Therapy with everolimus or sirolimus within the last 4 weeks
  Therapy Avastin within the last 4 weeks
8. Congenital haemorrhagic diathesis with need of perioperative treatment
9. Participation in another interventional trial with interference on intervention and primary outcome of this trial

**Table 1:** Details of exclusion criteria 3 and 4

Clinical exam	Radiological exam	Decision
The presence of a parastomal hernia or incisional hernia is *excluded* on the clinical exam	Not necessary	The patient **can** be included in the trial
Presence of a parastomal hernia > 8 cm or incisional hernia that would impede loop ileostomy reversal or placement of the mesh at the ileostomy site *confirmed* on clinical exam	Not necessary	The patient **cannot** be included in the P.E.L.I.O.N trial
Presence of a parastomal hernia or incisional hernia on clinical exam, but **unclear** whether: - Parastomal hernia is smaller or larger than 8 cm - Or if unclear whether incisional hernia impedes loop ileostomy reversal or placement of the mesh at the ileostomy site	Radiological exam (ultrasound or other) indicated	Inclusion/exclusion depends on radiological results

#### Eligibility criteria for trial sites and surgeons

- Only high-volume *trial sites* committing to include at least 10 patients per year will be chosen as trial sites.
- To ensure standardisation of the procedure and to minimise training effects, all *participating surgeons* must pass obligatory eLearning tutorials demonstrating the mesh-enforced loop ileostomy closure (experimental group) as well as non-mesh-based closure (control group) before participation in the trial.
- Furthermore, all *participating surgeons* must have performed a minimum of 10 loop ileostomy closures before participation in the trial. The number of previous ileostomy closures (<10 vs. ≥10) will be documented in the log staff for all participating surgeons.

### Who will take informed consent? {26a}

Patients scheduled for elective loop ileostomy closure are screened preoperatively. An authorised physician with a GCP training will inform the patient about the trial (visit 1). Patients are enrolled given their ability to understand the extent and nature of the trial as well as their written informed consent after detailed patient information.

### Additional consent provisions for collection and use of participant data and biological specimens {26b}

No biological samples will be collected during the trial.

## Interventions

### Explanation for the choice of comparators {6b}

Patients in both groups will receive ileostomy closure according to the current standard of care, i.e. direct fascia closure according to the small-stitch technique with a slowly absorbable suture. In addition, in the intervention group, the abdominal wall closure will be augmented with a non-absorbable, macro-pore light-weight mesh. Patients in the intervention group might therefore benefit from a potential decreased IH rate; however, the efficacy of the experimental intervention has not yet been shown. A real clinical equipoise exists between the two comparators (see the “Introduction” section).

### Intervention description {11a}

#### Experimental intervention

- The re-establishment of intestinal continuity will be achieved by either stapled or by hand-sewn anastomosis, according to the surgeon’s preference, and the bowel will be positioned back into the peritoneal cavity.
- Changing of surgical gloves must be performed before mesh implantation. Either after surgical stoma closure or, latest, before mesh placement.
- A retromuscular space is created by blunt dissection overlapping the suture line in cranio-caudal direction by 4–6cm. In the medial-to-lateral direction, the dissection is limited by the linea alba and linea semilunaris in case the stoma is placed within the rectus sheath. In case the stoma is outside the rectus sheath or below the linea arcuata (no posterior rectus sheath), a retromuscular space should be formed by dissection overlapping the future fascial suture line by 4–6cm in all directions.
- The posterior rectus sheath (if the stoma is within the rectus sheath) will be closed with a continuous polydioxanone suture (PDS Plus, USP 2-0) with tissue bites of 5 mm and intersuture spacing of 5 mm applied exclusively to the fascia omitting subcutaneous fat and muscle tissue (small-stitch technique, SST). In case the stoma is outside the rectus sheath or below the linea arcuata (no posterior rectus sheath), the posterior abdominal wall should be closed with a continuous polydioxanone suture (PDS Plus, USP 2-0) with tissue bites of 5 mm and intersuture spacing of 5 mm applied exclusively to the fascia omitting subcutaneous fat and muscle tissue (small-stitch technique, SST).
- The retromuscular space should be irrigated with 500 ml of sterile fluid (lavage) before mesh placement. The type of solution is at the discretion of the surgeon, e.g. saline solution (NaCl 0.9%), Ringer’s or any antiseptic solutions like polyhexanide (PHX), Lavanox or others can be used for this purpose as long as its application is approved for this purpose.
- A non-absorbable, macro-pore (pore size ≥ 1000μm [23] or effective porosity >0% [24]) light-weight monofilament (class Ia) or mixed structure (class Ic) mesh [24] is placed in the retromuscular position. The size of the mesh should overlap the suture line of the posterior rectus sheath by 4–6cm on all sides. In the medial-to-lateral direction, the mesh size might be limited by the linea alba and linea semilunaris (medial and lateral walls of the rectus sheath). To avoid any commercial interest of a single company, no specific mesh, but rather a well-described uniform CE-certified mesh class must be used in this trial. The safety of this class of meshes has already been shown in prospective trials in abdominal wall repair and ostomy formation [15].
- The mesh should be fixed to the posterior fascia tension-free with single knots if deemed necessary by the operating surgeon. The suture material is at the discretion of the operating surgeon.
- The rectus muscle should *not* be closed/approximated by sutures.
- The anterior rectus sheath will be closed in the same manner as the posterior sheath with a continuous polydioxanone suture (PDS Plus, USP 2-0) with tissue bites of 5 mm and intersuture spacing of 5 mm applied exclusively to the fascia omitting subcutaneous fat and muscle tissue (small-stitch technique, SST).
- Subcutaneous drains can be placed at the discretion of the operating surgeon.
- The skin will be closed according to the surgeon’s preference (suture, staples, pursestring).
- Further perioperative and postoperative treatment will be equal for both groups according to the respective local standard of care.

#### Control intervention

- The re-establishment of intestinal continuity will be achieved either by staples or by hand-sewn anastomosis, according to the surgeon’s preference, and the bowel will be positioned back into the peritoneal cavity.
- Changing of surgical gloves.
- The posterior rectus sheath (if the stoma is within the rectus sheath) will be closed with a continuous polydioxanone suture (PDS Plus, USP 2-0) with tissue bites of 5 mm and intersuture spacing of 5 mm applied exclusively to the fascia omitting subcutaneous fat and muscle tissue (small-stitch technique, SST). In case the stoma is outside, the rectus sheath or below the linea arcuata (no posterior rectus sheath), the posterior abdominal wall should be closed with a continuous polydioxanone suture (PDS Plus, USP 2-0) with tissue bites of 5 mm and intersuture spacing of 5 mm applied exclusively to the fascia omitting subcutaneous fat and muscle tissue (small-stitch technique, SST).
- The rectus muscle should *not* be closed/approximated by sutures.
- The *anterior* rectus sheath will be closed with a continuous polydioxanone suture (PDS Plus, USP 2-0) with tissue bites of 5 mm and intersuture spacing of 5 mm applied exclusively to the fascia omitting subcutaneous fat and muscle tissue (small-stitch technique, SST).
- Subcutaneous drains can be placed at the discretion of the operating surgeon.
- The skin will be closed according to the surgeon’s preference (suture, staples, pursestring).
- Further perioperative and postoperative treatment will be equal for both groups according to the respective local standard of care.

### Criteria for discontinuing or modifying allocated interventions {11b}

As P.E.L.I.ON. is investigating two surgical interventions, no criteria for discontinuing or modifying the allocated intervention are applicable.

### Strategies to improve adherence to interventions {11c}

As P.E.L.I.ON. is investigating two surgical interventions, strategies to improve adherence to the intervention are unnecessary as the patient cannot “withdraw” from the intervention during surgery.

### Relevant concomitant care permitted or prohibited during the trial {11d}

Further perioperative and postoperative treatment will be according to the local standard of care. This also includes the use of abdominal belts which is at the discretion of the operating surgeon. There will be no restrictions for additional treatments during the trial.

### Provisions for post-trial care {30}

For trials that are conducted according to the Medical Association’s professional code (Berufsordnung der Bundesärztekammer) § 15, there is no obligation for a specific trial liability insurance. However, patients will be insured against travel accidents for their follow-up visits (*Wegeversicherung*). No other specific post-trial care and no specific compensation to those who suffer harm from trial participation are implemented. Post-trial care will follow the clinical standard of care at the respective sites.

### Outcomes {12}

#### Primary outcome measure

An *incisional hernia* at the ileostomy closure site within 2 years of follow-up represents an outcome with the highest long-term impact for the patient and the healthcare system and is therefore selected as the primary endpoint. The duration of follow-up (2 years) is recommended by the EHS as the rate of IH increases after 1 year [25]. Furthermore, the primary endpoint is recommended by the EHS as it is clearly measurable and reproducible. Consequently, “any abdominal wall gap with or without a bulge in the area of a postoperative scar perceptible or palpable by clinical examination or imaging” is regarded as an IH*.*

Patients will be assessed for the primary endpoint at 12 and 24 months after the trial intervention. At these dates, patients will be examined by a clinician blinded for the trial intervention and by a radiologic examination performed by a blinded assessor. Radiologic exams allowed for assessment in the trial are for example sonography, computed tomography (CT) or magnetic resonance imaging (MRI) scans. To reduce radiation exposure, sonography is the preferred imaging method. No radiation-associated imaging should be initiated solely for the purpose of the P.E.L.I.O.N trial. However, results of radiation-associated imaging like CT scans that are performed for other indications (e.g. oncological follow-up) can be used in the trial. In this case, an additional ultrasound of the abdominal wall is not necessary. In case of conflicting results between clinical and radiologic exams, the radiologic imaging is decisive to increase sensitivity [25]. If only one of the two examinations is performed (i.e. either clinical or imaging), the result of this assessment will be used for analysis. As many patients included in this trial are expected to have an oncological indication and the included centres perform their oncological follow-up themselves, the loss to follow-up of patients is expected to be low. Possible results of the primary outcome assessment are listed in Table 2.

**Table 2:** Possible outcomes of clinical and radiological hernia assessment and outcome decisions in the P.E.L.I.O.N. trial

Clinical exam result	Imaging result	Primary endpoint
**Hernia**	Hernia	**Hernia**
**No hernia**	Hernia	**Hernia**
**Hernia**	No hernia	No hernia
**No hernia**	No hernia	No hernia
**Hernia**	Missing	**Hernia**
**No hernia**	Missing	No hernia
**Missing**	Hernia	**Hernia**
**Missing**	No hernia	No hernia

For patients who are unable or unwilling to attend the follow-up visits, a telephone follow-up is incorporated. As the patient-reported outcome questionnaire developed by Tastaldi et al. (Hernia Recurrence Inventory, HRI) was modified and will be used in all patients [26], it can be used as a screening tool during telephone visits, i.e. patients who are suspected to have an IH based on the questionnaire might be convinced to attend an outpatient visit, even if they were reluctant to do so before. Time-to-event analyses for the primary endpoint are not justified as it is highly dependent on the density of the follow-up schedule and clinical symptoms and our interest is whether a hernia occurs rather than when a hernia occurs.

#### Secondary outcome measures

1. Postoperative complications within 30 days according to the Dindo-Clavien classification (DCC) ≥3 [27]
  Number of surgical site occurrences (SSO) [28] defined as the sum of the following *individual* secondary endpoints:
2. *Superficial or deep surgical site infections* (SSIs) within 1 year after index operation according to the CDC definition [29]. Follow-up for this endpoint will be 1 year as defined by the CDC in case of foreign body placement [29]. Organ-space SSIs are excluded in this measurement as they are independent of the abdominal wall closure technique, but rather depend on the underlying surgery. Furthermore, they are infrequent after ileostomy reversal. In addition, clinically relevant organ-space SSIs (defined as Dindo-Clavien ≥ grade 3) will be recorded in the overall complication rate according within 30 days
3. Rate of *wound seromas* at former ostomy site within 30 days after index operation. Seroma will be defined as a collection of serous fluid in a dead space, which can either be in situ or leaking through a wound [30]. Assessment can be by clinical or radiologic examination
4. Rate of *wound hematomas* at former ostomy site within 30 days after index operation. Hematomas are defined as an accumulation of blood in the wound area, which warrants (bedside) surgical exploration and intervention [31]
5. *Enterocutaneous fistulas* within 24 months after index operation [28]
6. Number of patients undergoing *incisional hernia repair* at the site of ileostomy closure within 24 months after the index operation
7. Number of *revision surgeries* because of complications related to ileostomy closure within 24 months after the index operation
8. *Chronic postoperative pain* in the ileostomy closure site during follow-up measured via the Pain Questions of the modified EHS-QoL questionnaire at 12 and 24 months [32]
9. *Health-related quality of life* at baseline, 12 and 24 months after index operation according to the EHS-QoL questionnaire [32]
10. *Modified Hernia Recurrence Inventory (HRI)* at 12 and 24 months [26]

### Participant timeline {13}

**Fig. 1:**
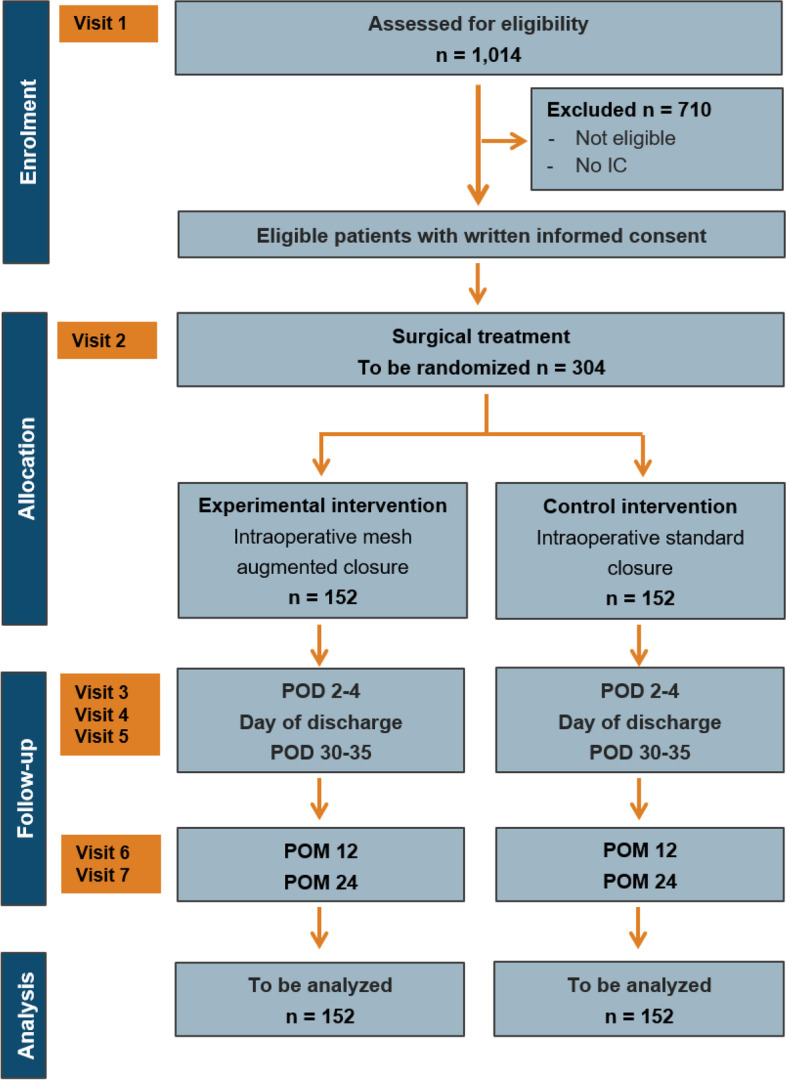
Flow chart. IC, informed consent; POD, postoperative day; POM, postoperative month

Patients scheduled for elective loop ileostomy closure are screened preoperatively (visit 1) (Fig. 1). Patients are enrolled given their ability to understand the extent and nature of the trial as well as their written informed consent after detailed patient information. All inclusion criteria and no exclusion criteria must be fulfilled. Baseline data are collected during screening/baseline visit. Included patients are randomised during surgery (visit 2). The ostomy closure will be performed as randomised. Follow-up visits will be on postoperative days 2–4, at discharge and on days 30–35 (visits 3–5) for evaluation of secondary endpoints. In addition, 12 and 24 months (visits 6–7) after surgery patients are planned for follow-up to evaluate primary and secondary outcome parameters. In case the patient is unable to attend clinical visits 6–7, a telephone evaluation will be performed. If the interview reveals a strong indication for the presence of an incisional hernia, the patient will be once again asked to present to the outpatient clinic for clinical and ultrasound evaluation. Protocol deviations will be assessed during the entire study period. An overview of the trial visits and collected data can be found in Table 3.

**Table 3:** Trial visits

Activity	Visit 1 (screening)	Visit 2 (surgery, randomisation)	Visits 3–5 (PODs 2–4, discharge, POD 30–35)	Visits 6–7 (POMs 12, 24)
**Inclusion/exclusion criteria**	x			
**Informed consent**	x			
**Medical history**	x			
**Clinical examination**	x			
**Surgery**		x		
**Randomisation**		x		
**Incisional hernia assessment**^**a**^				x
**Assessment of postoperative morbidity**^**b**^			x	
**Assessment of superficial and deep SSI**^**c**^			x	x (not at POM 24)
**Assessment of wound seroma**			x	
**Assessment of wound hematoma**			x	
**Assessment of enterocutaneous fistula**			x	x
**Incisional hernia repair at the site of ileostomy**			x	x
**Assessment of revision surgery**			x	x
**Pain assessment**^**d**^	x			x
**Health-related quality of life assessment**^**e**^	x			x
**Modified Hernia Recurrence Inventory**^**f**^				x

*POD* postoperative day, *POM* postoperative month, *SSI* surgical site infection

^a^Via blinded assessor: clinical and radiologic assessment

^b^According to Dindo-Clavien classification ≥ 3

^c^Via blinded assessor, according to CDC definition [29]

^d^Measured via the Pain Questions of the EHS-QoL questionnaire [32]

^e^According to the modified EHS -QoL questionnaire [32]

^f^Using the modified Hernia Recurrence Inventory [26]

### Sample size {14}

Sample size calculation is based on the rate of the primary endpoint (IH rate) within 24 months after surgery. Based on the assumption that the percentage of patients developing an IH in the control group is 30% (4–7), we hypothesise a reduction to 15% in the intervention arm (conservative reduction rate below that reported in the above-mentioned trials). Consequently, a sample size per group of 121 patients is needed for the group comparison by the chi-squared test to achieve 80% power in detecting this difference in IH rate at a two-sided level of significance of 5%. It is assumed that using a logistic regression model adjusting for IH risk (low-risk vs. high-risk patients: BMI ≥ 27) and including centre as a random effect in the primary analysis will lead to less unexplained variance and thus to an increased power.

The intercurrent event re-laparotomy affecting the loop ileostomy closure site and death without prior observation of IH are expected to be very rare, with just a few patients each. Based on previous trials, to account for the loss of information, a rate of 20% of dropouts, loss to FU and relevant ICEs (handled with the hypothetical strategy) is considered. Therefore, a total of 304 patients (152 per group) will be randomised. The other ICEs are ignored in the primary estimand and not accounted for in the sample size calculation. Calculation was performed using PASS 16.0.12. Only high-volume trial sites committing to include at least 10 patients per year will be chosen as trial sites.

### Recruitment {15}

To enrol the required number of patients in the planned recruitment period, 13 trial sites will participate in this trial. The trial will be performed by the Clinical Trial Network of the German Society of Surgery (CHIR-Net, www.chir-net.de). The CHIR-Net has successfully performed trials with similar indications and recruitment rates. All trial sites have already successfully participated in surgical clinical trials and have the necessary expertise, equipment and personnel to perform this trial. Participating centres have an estimated annual case load greater *n*=500, ensuring that recruitment should be feasible within the planned recruitment time. It is aimed to minimise loss to follow-up by a pragmatic trial design with high-external validity and financial reimbursement of travel costs for patients covering additional costs for trial-specific visits.

In order to minimise loss to follow-up, the following measures are being implemented:

- A pragmatic trial design with high-external validity, meaning that trial sites will have little problems to adapt their standard clinical workflow to the follow-up visits
- Financial reimbursement of travel costs for patients covering additional costs for trial-specific visits
- All trial sites are trained in performing surgical clinical trials (CHIR-Net) and have successfully participated in similar trials

Only high-volume trial sites committing to include at least 10 patients per year will be chosen as trial sites.

## Assignment of interventions: allocation

### Sequence generation {16a}

To ensure equal distribution of patient characteristics, randomisation will be used. Allocation of treatments will be performed using a web-based randomisation tool (www.randomizer.at). Block-wise randomisation will be conducted. Randomisation will be stratified by centre.

### Concealment mechanism {16b}

Allocation concealment will be ensured as the result of the randomisation will be unknown to the study team until the patient has been randomised through the web-based tool. Block size will be kept confidential to the study team. Randomisation will be performed in the operation theatre, after the closure of the posterior rectus sheath/posterior fascia. This prevents potential bias by different intraoperative techniques. Before randomisation, the surgeon needs to rule out an infected/septic surgical site (risk for surgical site occurrences (SSO) of grade 4 according to Ventral Hernia Working Group (VHWG) classification) [28].

### Implementation {16c}

Randomisation will be performed by a study team in the operation theatre. Names of this team member and the operating surgeon are noted as these team members are unblinded. They will not be involved in the outcome assessment of the trial.

## Assignment of interventions: blinding

### Who will be blinded {17a}

Blinding will be performed according to the evidence-based guidelines published by Probst et al. [33].

A. Patients are blinded as they are under general anaesthesia during the trial intervention. Neither the discharge letter nor the operation report will contain any information regarding group allocation. This will be monitored by the clinical monitor.
B. Surgeons performing the operation are unblinded to the trial intervention. To minimise performance bias, randomisation is performed after the closure of the posterior wall of the abdomen.
C. For blinded outcome assessment, the person performing randomisation and the surgical team conducting the control/experimental intervention (“unblinded” study members) will be documented. All outcome assessments must be performed by blinded clinical assessors *not* being part of this group. This must be documented and will be verified by the clinical monitor.
D. The trial statistician is blinded as she/he has no access to unblinded data during the study. Furthermore, the statistician will perform according to a pre-defined statistical analysis plan which will be finished prior to database closure.
E. For primary endpoint evaluation, a clinical as well as radiological examination needs to be performed. Clinical outcome assessment must be performed by a surgeon. Blinding the radiologic examination can be achieved easily, as these physicians are not part of the prior trial interventions or the clinical trial centre. For clinical evaluation, the same prerequisites apply as outlined above, i.e. surgeons performing the clinical evaluation must be independent of the “unblinded” trial team.

### Procedure for unblinding if needed {17b}

Unblinding can be performed if medically indicated by the treating surgeon with the help of an unblinded study team member. Unblinding will be documented and reported to the steering group of the trial.

## Data collection and management

### Plans for assessment and collection of outcomes {18a}

The trial will be performed by the Clinical Trial Network of the German Society of Surgery (CHIR-Net, www.chir-net.de). The CHIR-Net has successfully performed trials with similar indications and recruitment rates [34–36]. All trial sites have already successfully participated in surgical clinical trials and have the necessary expertise, equipment and personnel to perform this trial.

### Plans to promote participant retention and complete follow-up {18b}

In order to minimise loss to follow-up, the following measures have been implemented:

- A pragmatic trial design with high-external validity, meaning that trial sites will have little problems to adapt their standard clinical workflow to the follow-up visits
- Financial reimbursement of travel costs for patients covering additional costs for trial-specific visits
- All trial sites are trained in performing surgical clinical trials (CHIR-Net) and have successfully participated in similar trials
- Only high-volume trial sites committing to include at least 10 patients per year will be chosen as trial sites

### Data management {19}

All protocol-required information collected during the trial must be entered by the investigator, or designated representative, in the eCRF. The investigator, or designated representative, should complete the eCRF pages as soon as possible after information is collected, preferably on the same day that a trial subject is seen for an examination, treatment or any other trial procedure. Any outstanding entries must be completed immediately after the final examination. An explanation should be given for all missing data. Further protocol deviations are explicitly asked for in the eCRF and have to be described by trial centres. The completed eCRF must be reviewed and signed by the investigator named in the trial protocol or by a designated sub-investigator.

The IMBI is responsible for the data management within the trial. The study data will be collected and managed using REDCap (Research Electronic Data Capture) [37], a secure, web-based data capture application hosted at the IMBI. To assure a safe and secure environment for the data acquired, data transmission is encrypted with secure socket layer (SSL) technology. Only authorised users are able to enter or edit data, and the access is restricted to data of the patients in the respective centre. All changes to data are logged with a computerised timestamp in an audit trail. All data will be pseudonymised. To guarantee high data quality, data validation rules will be defined in a data validation plan. Completeness, validity and plausibility of data will be checked in time of data entry (edit-checks) and using validating programmes, which will generate queries. If no further corrections are to be made in the database, eCRF data will be locked. Data will finally be downloaded and used for statistical analysis. All data management procedures will be conducted according to written defined standard operating procedures (SOPs) of the IMBI that guarantee an efficient conduct complying with GCP. At the end of the study, the data will be transformed into different data formats (e.g. csv-files) for archiving and to ensure that it can be re-used.

### Confidentiality {27}

The data protection concept stipulates that patients are only included in the study after they have been informed and after having signed the informed consent form (ICF). They will be informed as to the strict confidentiality of their data, but that their medical records may be reviewed for trial purposes by authorised individuals other than their treating physician. It is the responsibility of the investigator to maintain patients’ confidentiality. During the trial, patients will be identified solely by means of their individual identification code, and participating patients’ data will be recorded only in pseudonymised form. Trial-specific documents will be stored in accordance with local data protection law/ICH-GCP guidelines and will be handled in the strictest confidence. For the protection of these data, organisational procedures are implemented to prevent the distribution of data to unauthorised persons. Each trial site will maintain a personal subject identification list (screening numbers with the corresponding subject names) to enable records to be identified. The patients’ data will be transferred in a pseudonymised form from the trial centre to cooperating partners (coordinating investigator, data management). Names and all confidential data of participating patients will be handled in line with the obligations of medical secrecy, the European General Data Protection Regulation GDPR (Datenschutzgrundverordnung, DSGVO), the Federal Data Protection Act (Bundesdatenschutzgesetz) and the state Data Protection Act (Landesdatenschutzgesetz). Third parties have no access to original documents. After completion of the trial, data collected during the study will be kept on file for 10 years. It is guaranteed that the data protection provisions are followed.

### Plans for collection, laboratory evaluation and storage of biological specimens for genetic or molecular analysis in this trial/future use {33}

No plans for collection, laboratory evaluation and storage of biological specimens for genetic or molecular analysis are implemented in the P.E.L.I.O.N. trial.

## Statistical methods

### Statistical methods for primary and secondary outcomes {20a}

In the Addendum to the ICH E9 guideline, the estimands framework is recommended as a clear and transparent definition of “what is to be estimated” (International Council for Harmonization, 2019). In the following, the primary estimand corresponding to the primary objective is described:

- *Treatment*: Abdominal wall closure during loop ileostomy closure with a continuous slowly absorbable suture reinforced with a retromuscular non-absorbable, macro-pore (pore size ≥ 1000μm or effective porosity >0%) light-weight monofilament (class Ia) or mixed structure (class Ic) mesh (experimental arm) vs. abdominal wall closure during loop ileostomy closure with a continuous slowly absorbable suture
- *Population*: The targeted population is defined through the inclusion and exclusion criteria
- *Variable*: Incisional hernia (IH) within 24 months after intervention as defined by the European Hernia Society
- *Intercurrent events (ICEs):*
  - Re-laparotomy affecting the loop ileostomy closure site without prior observation of IH: This event is exceedingly unlikely because the presence/absence of an IH will be observed at the time of re-laparotomy. Nevertheless, re-laparotomy affecting the loop ileostomy closure site without prior observation of IH presents a potential ICE. This is assumed to be independent of the intervention but influences the probability of IH occurrence. Since the estimate of interest is the treatment effect as if no re-laparotomy had been performed, the later occurrence (or lack thereof) of IH will not be considered. Instead, the primary endpoint will be imputed equivalently to patients who dropped out or were lost to follow-up, i.e. by multiple imputation. This handling conforms to a hypothetical strategy.
  - Surgical site infections: Will not be considered, reflecting a treatment policy approach.
  - Treatment switching: Will not be considered, reflecting a treatment policy approach. The risk of switching patients has been minimised through the choice of inclusion and exclusion criteria. However, switching may be needed due to clinical reasons getting obvious during surgery (e.g. mesh implantation technically not possible, mesh implantation necessary due to parastomal hernia)
  - Death without prior observation of IH: Assuming independence of closure with or without mesh and death, the event is handled using the hypothetical strategy
  - Oncological systemic treatment therapy before or after the surgical intervention (e.g. chemotherapy): Systemic therapy is assumed to be unlikely to affect the primary endpoint. Reflecting a treatment policy, adjuvant therapy will be ignored
- *Summary measure: Differences in incisional hernia rates*

The hypotheses to be assessed in the primary efficacy analysis are as follows: 𝐻_0_: 𝑝_T_ = 𝑝_C_ and 𝐻_1_: 𝑝_T_ ≠ 𝑝_C_, where 𝑝_T_ and 𝑝_C_ denote the IH rates in the intervention and control groups, respectively.

The confirmatory analysis of the primary efficacy endpoint corresponds to the primary estimand defined in chapter 1.5. and will be done based on the full analysis set (FAS) according to the intention-to-treat principle. The FAS consists of all randomised patients and the patients will be considered in the group randomised to. The level of significance is set to 5% (two-sided).

The IH rates will be compared via a mixed logistic regression model including the factors treatment group and IH risk (high vs. low) as fixed effects and centre as a random effect. As the number of centres is relatively large in relation to the sample size, inclusion of the centre as a random effect instead of a fixed effect is indicated. Differences between centres cannot be ruled out but are of no special interest in the primary analysis; inclusion of centre as the random effect is recommended [38]. Besides this, the interpretation is more straightforward because “[…] in the context of RCTs, centre effects can be regarded as a nuisance parameter, as the primary goal is to estimate the treatment effect”. For the structure of the covariance matrix of the random intercept of centre, a variance component structure is used allowing for different variability within each centre while assuming that centres are independent. Missing data for the primary outcome variable due to dropout or loss to follow-up are assumed to be at least “missing at random” and will be replaced by using multiple imputation. Occurrence of IH after the ICE re-laparotomy affecting the loop ileostomy closure site and death without prior observation of IH will be imputed the same way. Information whether a patient was event-free regarding the occurrence of an IH before loss-to-follow-up, dropout or the respective ICEs will be included into the multiple imputation procedure. This will be done by imputing the primary endpoint “incisional hernia within 24 months” (yes/no) using the variable “incisional hernia within 12 months” (yes/no) besides treatment group, IH risk (high vs. low) and centre using the fully conditional specification (FCS) and the discriminant function method. ICEs that are handled using a treatment policy strategy will be ignored in the primary analysis. The proportion of each ICE will be described by group to support the interpretation of the primary effect estimate.

Sensitivity analyses regarding the imputation method and the underlying assumptions will be performed. These include applying a pattern mixture model under the assumption of a “missing not at random” data mechanism, as well as best- and worst-case scenarios to assess the robustness of the results.

Within a sensitivity analysis, oncologic treatment will be included into the logistic regression model for the primary endpoint as an additional covariate.

The complementary estimands will be estimated using the same primary analysis strategy as used for the primary estimand with the following differences: To estimate the first complementary estimand, the primary endpoint in the switched patients will not be taken as observed but will be imputed in the group as treated. The second complementary estimand will be estimated based on all patients treated as randomised (treatment switcher will be excluded). In the following, supplementary analyses for the primary estimand are described.

The same test as in the primary analysis will be conducted in the per-protocol set (PP, based on those patients without major protocol violation). Failure to respect the exclusion criteria or to follow the technical aspects of the assigned study group (suture material, stitch technique, used mesh) will be considered as major protocol violations. Analyses in the PP set are known to be biased and do not correspond to any estimand and thus have to be interpreted with great caution. Based on the FAS set, a complete case analysis will be performed without imputation of missing data.

All baseline variable and secondary outcomes will be evaluated descriptively, and descriptive *p-*values are reported together with 95% confidence intervals for the corresponding effects. As in the primary analysis, secondary endpoints will be assessed by a mixed regression model including the treatment group as a fixed effect and centre as a random effect. For each endpoint, relevant covariates which will be included in the analysis are pre-defined.

The safety analysis includes the calculation of frequencies and rates of complications (specific secondary outcome parameters) together with 95% confidence intervals. This analysis will be based on the safety set that contains all randomised patients in the group as treated.

All analyses will be done using SAS version 9.4 or higher.

### Interim analyses {21b}

Due to the long observation period for the primary endpoint, an interim analysis would only be possible using a short-term endpoint as a surrogate. However, no reliable short-term endpoint is available. Therefore, no interim analyses are planned for the present trial. However, the DSMB will oversee unblinded data on a regular basis.

### Methods for additional analyses (e.g. subgroup analyses) {20b}

Pre-specified subgroup analysis will be performed in the FAS population for the rate of IH in the following subgroups: (a) body mass index (< 27 vs. ≥ 27), (b) neoadjuvant therapy (yes/no), (c) COPD vs. non-COPD, (d) previous laparotomy vs. previous minimal invasive surgery and (e) history of hernia/hernia repair vs. no history of hernia/hernia repair.

Furthermore, uni- and multivariate analyses using two-level binary logistic regression models will be performed to find risk factors for IH. Factors that will be included in the model (besides the fixed factors treatment group and IH risk, and the random factor centre which are already used in the primary model) are age, BMI, sex, presence/absence of diabetes mellitus with chronic complications, corticosteroid use, preoperative (radio)chemotherapy, renal disease, smoking, previous laparotomies, COPD, cardiovascular disease, history of hernia or hernia repair, history of abdominal aortic aneurysm, ASA classification, occurrence of SSI, postoperative complication (Dindo-Clavien), seroma formation, hematoma formation and length of hospital stay.

Furthermore, the treatment effect will be assessed descriptively within several subgroups of interest to identify potential prognostic and predictive factors.

### Methods in analysis to handle protocol non-adherence and any statistical methods to handle missing data {20c}

For patients with incomplete follow-up, missing data will be shown as missing on appropriate tables and listings. Imputation of main secondary outcomes will be performed.

### Plans to give access to the full protocol, participant-level data and statistical code {31c}

The full protocol is accessible with this publication. Participant-level data will be available anonymised after the publication of the final results of the study.

## Oversight and monitoring

### Composition of the coordinating centre and trial steering committee {5d}

The trial will have a steering committee consisting of the trial statistician (AS), three clinical experts (AM, SM, DW) and a patient representative (EG). The steering committee will supervise the conduct of the trial and will issue recommendations for early termination, modifications or continuation of the trial, if necessary.

### Composition of the data monitoring committee, its role and reporting structure {21a}

The trial’s Data Safety and Monitoring Board (DSMB) will be composed of two independent clinical experts in the field of hernia surgery. Furthermore, an independent statistician will be part of the DSMB. The DSMB members will receive a written report twice a year and should advise whether to continue, modify or stop the trial based on the rate and severity of specific intervention-associated complications (see secondary endpoints SSO) and of major (≥ grade 3) postoperative complications according to Dindo-Clavien (within 30 days after ileostomy closure). Furthermore, they should review adherence to protocol especially for the trial-relevant primary outcome measure and will inform the coordinating investigator about relevant imbalances between groups. DSMB members will also be asked to give advice on whether results from other relevant trials justify continuing recruitment of further patients. The working procedures of the DSMB will be recorded in the DSMB charter of this trial.

### Adverse event reporting and harms {22}

Since trial interventions in P.E.L.I.O.N are medical routine, and the trial is conducted under the Medical Association’s professional code (Berufsordnung der Bundesärztekammer) §15, there is no need to record every adverse event. Instead of documenting serious adverse events (SAE), the following secondary endpoints will be used to assess safety in the P.E.L.I.O.N trial:

1. Postoperative complications within 30 days according to the Dindo-Clavien classification (DCC) ≥3 [27]
2. Superficial or deep surgical site infections (SSIs) within 1 year after index operation according to the CDC definition [29]
3. Rate of wound seromas at former ostomy site within 30 days after the index operation
4. Rate of hematomas at former ostomy site within 30 days after the index operation
5. Enterocutaneous fistulas within 24 months after the index operation [28]
6. Number of revision surgeries because of complications related to ileostomy closure within 24 months after the index operation

### Frequency and plans for auditing trial conduct {23}

Clinical monitoring will be performed regularly by the independent monitoring department of the *Zentrum für klinische Studien* (ZKS) Ulm according to its standard operating procedures. A risk-based monitoring strategy will be conducted based on patient safety, protocol adherence and data validity and a study-specific pre-defined monitoring manual. On-site monitoring will focus on patient informed consent and safety, surgical procedures, blinding and correct recording and documentation of primary and secondary endpoints by source data verification. Informed consent and the primary endpoint will be checked in all patients. To verify blinding, discharge and surgery reports of a specific number of patients will be checked to verify that group allocation is not mentioned.

Furthermore, for a specific number of long-term postoperative visits (12 and 24 months postoperative), monitoring will confirm that primary outcome assessment is performed by blinded outcome assessors. For other items, the proportion of source data verification will be at least 20% but can be increased according to centres’ experience and needs. The frequency of monitoring visits will be determined depending on recruitment numbers and individual performance of each centre based on feedback from project and data management and according to the risk-based monitoring approach.

The steering committee will meet at least twice a year. Furthermore, the DSMB members will receive a written report twice a year and should advise whether to continue, modify or stop the trial based on the rate and severity of specific intervention-associated complications and of major (≥ grade 3) postoperative complications according to Dindo-Clavien (within 30 days after ileostomy closure). The decision to discontinue recruitment will be made by the coordinating investigator and the steering committee after the DSMB gave its recommendation. The coordinating investigator and/or the steering committee may call upon the DSMB in case a safety issue arises during the course of the trial.

### Plans for communicating important protocol amendments to relevant parties (e.g. trial participants, ethical committees) {25}

Any amendment to the protocol will be communicated to the participating trial sites immediately by email. Furthermore, regular investigator meetings will be held during the twice yearly CHIR-Net meetings to resolve questions and discuss the progress of the trial. Trial registries, REC/IRBs, will be contacted by the project management and the steering group.

## Dissemination plans {31a}

Trial results will be communicated to participating trial sites prior to publication. The final report will be reviewed by all trial sites. Results will be made available in an open access journal. Results will be communicated to appropriate patient organisations like the ILCO e.V. Trial results will be presented at an international conference.

## Discussion

Given the high rate of IH after loop ileostomy closure with its associated morbidity, loss of health-related quality of life and costs, prophylactic measures after ostomy closure are urgently needed. Prophylactic mesh implantation at the time of surgical stoma reversal has shown to be a promising and safe method to prevent IH formation. However, the efficacy of this method has not yet been investigated in a large multicentre RCT with adequate external validity. P.E.L.I.O.N will fill this evidence gap. The aim of the PELION trial is to evaluate the efficacy of prophylactic mesh reinforcement after loop ileostomy closure in decreasing the rate of IH versus standard closure alone. Recruitment of patients has started in October 2022 and is going as projected.

Besides P.E.L.I.O.N, four planned randomised controlled trials are registered in available international registries, all with relevant weaknesses in the trial design, making interpretation and generalisation of the results doubtful (see Table 4). Therefore, a further multicentric randomised controlled trial with sufficient external validity is warranted to support or abandon the use of mesh reinforcement of the abdominal wall during loop ileostomy closure.

**Table 4:** Planned studies currently registered

Study name/registration number	Potential methodical weaknesses
ILEOCLOSE/NCT02226887	Inadequate follow-up (12 months), absorbable mesh (biomaterial, not recommended by EHS), inadequate mesh size (1cm wide stripe), inlay mesh position (not recommended by EHS), underpowered sample size
ILEOMESH/NCT02896686	Single-blinded trial. Inadequate follow-up (12 months), inadequate mesh size (3cm wide stripe), onlay mesh position, underpowered sample size
MEMBO/NCT02576184	Three arms, two experimental arms (high risk of bias), absorbable mesh (biological) in one arm and obsolete mesh material (polyester) in the other, only rectal cancer patients included
LISTO/NCT02669992	Primary endpoint postoperative bowel obstruction not IH, four arms (high risk of bias) with inadequate participants’ number, mesh size not defined, only rectal cancer patients included

## Trial status

The current protocol number is 1.1, May 12, 2022.

Recruitment has started in October 2022.

Recruitment is planned to be completed at the beginning of 2024.

## Supplementary Information

**Additional file 1.** List of study sites.

**Additional file 2.** Study specific instruments of the P.E.L.I.O.N. trial.

## Data Availability

It is planned to make anonymous trial data on which scientific publications are based and all anonymous primary data publicly available for re- and meta-analyses after completion of the trial in an appropriate repository.

## Abbreviations

AE: Adverse event
eCRF: Electronic case report form
CDC: Centres of Disease Control and Prevention
CHIR-Net: Clinical Trial Network of the German Association of Surgery
DCC: Dindo-Clavien classification
DRKS: Deutsches Register klinische Studien (German Clinical Trials Register)
DSMB: Data Safety Monitoring Board
EC: Ethics Committee
EHS-QoL: European Hernia Society Quality of Life questionnaire
FAS: Full analysis set
FPI: First patient in
FU: Follow-up
GCP: Good Clinical Practice
HRI: Hernia Recurrence Inventory
ICE: Intercurrent event
ICH: International Conference on Harmonization of Technical Requirements for Registration of Pharmaceuticals for Human Use
IH: Incisional hernia
IMBI: Institute of Medical Biometry
ITT: Intention-to-treat
ISF: Investigator site file
LPI: Last patient in
LPO: Last patient out
POD: Postoperative day
POM: Postoperative month
QoL: Quality of life
RCT: Randomised controlled trial
SAE: Serious adverse event
SDGC: Studienzentrum der Deutschen Gesellschaft für Chirurgie Study Center of the German Society of Surgery
SOP: Standard operating procedure
SSO: Surgical site occurrence
V: Visit
ZKS: Zentrum für Klinische Studien (Center for Clinical Studies)
TMF: Trial Master File
