# Supplementary material for: Prophylactic effect of retromuscular mesh placement during loop ileostomy closure on incisional hernia incidence—a multicentre randomised patient- and observer-blind trial (P.E.L.I.O.N trial)
Source: Trials. 2023 Feb 1;24:76. doi: 10.1186/s13063-023-07089-3 (PMC9890770; doi:10.1186/s13063-023-07089-3)
Supplement: Supplementary file 1 — Additional file 1. List of study sites. [file 13063_2023_7089_MOESM1_ESM.docx]

| **Zentrum** | **Trial site** | **PI** |
| --- | --- | --- |
| Universitätsklinikum Freiburg  Department Chirurgie  Klinik für Allgemein- und Viszeralchirurgie | Department of General and Visceral Surgery  Department of Surgery, Medical Center  University of Freiburg | Dr. med. Catharina Ruf |
| Asklepios Klinik Langen  Klinik für Allgemein-, Viszeral- und Thoraxchirurgie | Asklepios Hospital Langen  Department of General, Visceral and Thoracic surgery | Dr. med. Sabine Schneider |
| Universität Heidelberg  Medizinische Fakultät Mannheim  Chirurgische Klinik | Department of Surgery, University Medical Centre Mannheim (UMM), Medical Faculty Mannheim, University of Heidelberg | PD Dr. med. Florian Herrle |
| Klinikum der Ludwig-Maximilians-Universität München  Klinik für Allgemein, Viszeral- und Transplantationschirurgie  Campus Großhadern | Klinikum der Ludwig-Maximilians-Universität München  Department of General, Visceral and Transplantation Surgery, University of Munich | PD Dr. med. Markus Albertsmeier |
| Klinik und Poliklinik für Chirurgie  Klinikum rechts der Isar der  Technischen Universität München | Department of Surgery  University Hospital Rechts der Isar  Technical University Munich | Prof. Dr. med. Daniel Reim |
| Pius-Hospital Oldenburg  Klinik für Allgemein- und Viszeralchirurgie  Universitätsklinik für Viszeralchirurgie | Pius Hospital Oldenburg  Clinic for General and Visceral Surgery  University Clinic for Visceral Surgery | Dr. med. Ralf Heinzel |
| GRN-Klinik Sinsheim  Allgemein- und Viszeralchirurgie | GRN-Klinik Sinsheim  General and Visceral Surgery | Dr. med. Matthias Hassenpflug |
| Universitätsklinikum Tübingen  Universitätsklinik für Allgemeine, Viszeral- und Transplantationschirurgie | University Hospital Tübingen  University Hospital for General, Visceral and Transplant surgery | PD Dr. Robert Bachmann |
| Bundeswehr Krankenhaus Ulm  Klinik für Allgemein-, Viszeral- und Thoraxchirurgie | Bundeswehr Hospital Ulm  Department of General, Visceral and Thoracic surgery | Matthias Zieringer |
| Universitätsklinikum Ulm  Klinik für Allgemein- und Viszeralchirurgie | University Hospital Ulm  General and Visceral Surgery | Prof. Dr. med. Andre Mihaljevic |
| Universitätsklinikum Würzburg  Klinik und Poliklinik für Allgemein-, Viszeral-, Transplantations-, Gefäß- und Kinderchirurgie | University Hospital Würzburg  Department of General, Visceral, Vascular, and Pediatric Surgery | PD Dr. med. Johan Lock |
