# Supplementary material for: Prophylactic effect of retromuscular mesh placement during loop ileostomy closure on incisional hernia incidence—a multicentre randomised patient- and observer-blind trial (P.E.L.I.O.N trial)
Source: Trials. 2023 Feb 1;24:76. doi: 10.1186/s13063-023-07089-3 (PMC9890770; doi:10.1186/s13063-023-07089-3)

**Supplement 2 – Study specific instruments of the P.E.L.I.O.N. trial**

Risk for Surgical Site Occurrencies (SSO) according to Ventral Hernia Working Group (VHWG). Taken from Breuing K, Butler CE, Ferzoco S, Franz M, Hultman CS, et al. Surgery. 2010 Sep;148(3):544–58.


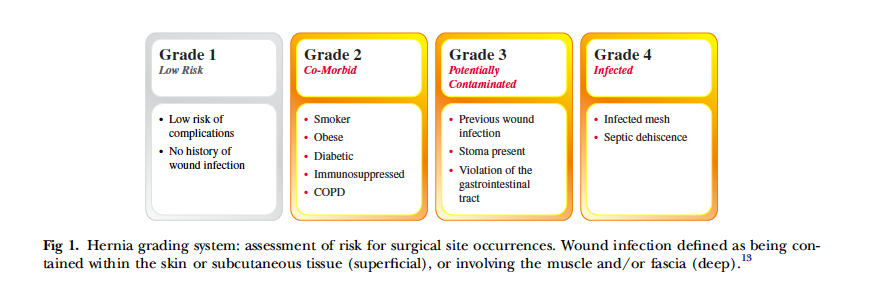


EHS-QoL questionnaire. Muysoms FE, et al. Surgery. 2016 Nov;160(5):1344–57


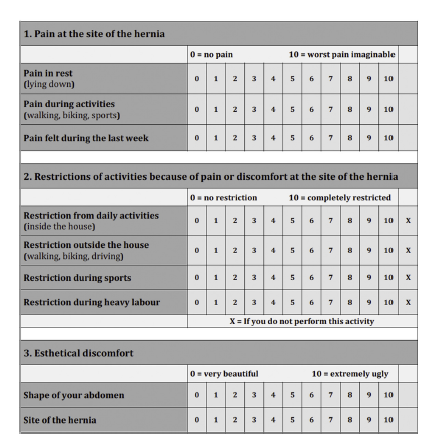


Hernie Recurrence Inventory. Tastaldi L, et al. Hernia. 2020 Feb 1;24(1):127–35.


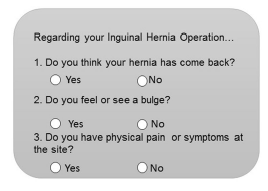


Charlson comorbidity index in its updated version described by Quan et al. (39)


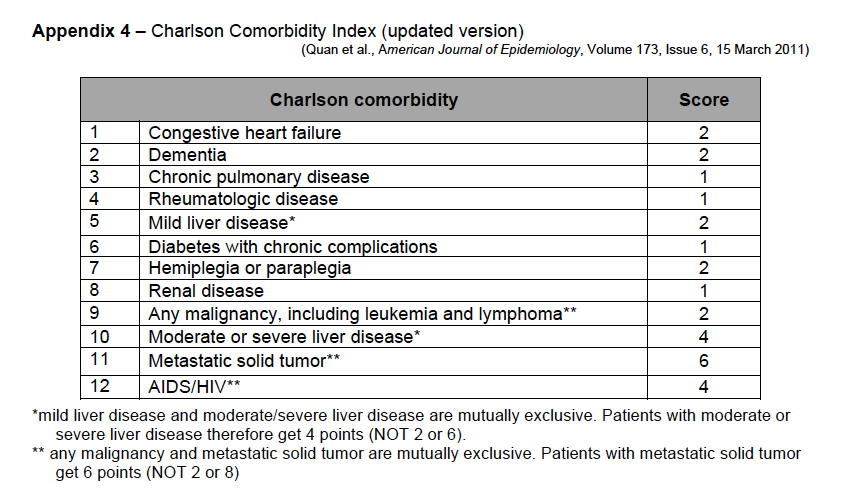

Supplement: Supplementary file 2 — Additional file 2. Study specific instruments of the P.E.L.I.O.N. trial. [file 13063_2023_7089_MOESM2_ESM.docx]
